# Herbivorous damselfishes expand their territories after causing white scars on *Porites* corals

**DOI:** 10.1038/s41598-020-73232-8

**Published:** 2020-09-30

**Authors:** Hiroki Hata, Shota Takano, Hiroyuki Masuhara

**Affiliations:** Graduate School of Science and Engineering, Ehime University, 2-5 Bunkyo, Matsuyama, Ehime 790-8577 Japan

**Keywords:** Behavioural ecology, Ecology

## Abstract

Turf algae become the most abundant benthic group on coral reefs after mass coral bleaching. By defending feeding territories, damselfishes enhance the growth of turf algae in so-called algal farms and affect coral communities both directly and indirectly. We found several white scars (i.e., bite lesions) on massive *Porites* colonies around feeding territories. In this study, we examined the occurrence of white scars on corals and their function in coral–algal competition at the boundaries between algal farms of two damselfish species*—*the intensive farmer *Stegastes nigricans*, and the intermediate farmer *S. lividus—*and adjacent *Porites* corals for 3 years around Okinawa Island, Japan. White scars occurred on *Porites* colonies only adjacent to the territories of both damselfish species. Of the white scars on corals around *S*. *nigricans* territories, 73% of the area was covered by algae within 2 weeks, while the remaining was re-covered by *Porites* tissues. The coral–algal boundaries encroached further into areas of coral when the area of white scars were larger. These results suggest that both intensive and intermediate farmers bite adjacent *Porites* colonies causing white scars on corals, and expand their territories onto corals using algae-covered white scars as stepping stones.

## Introduction

**Figure 1:**
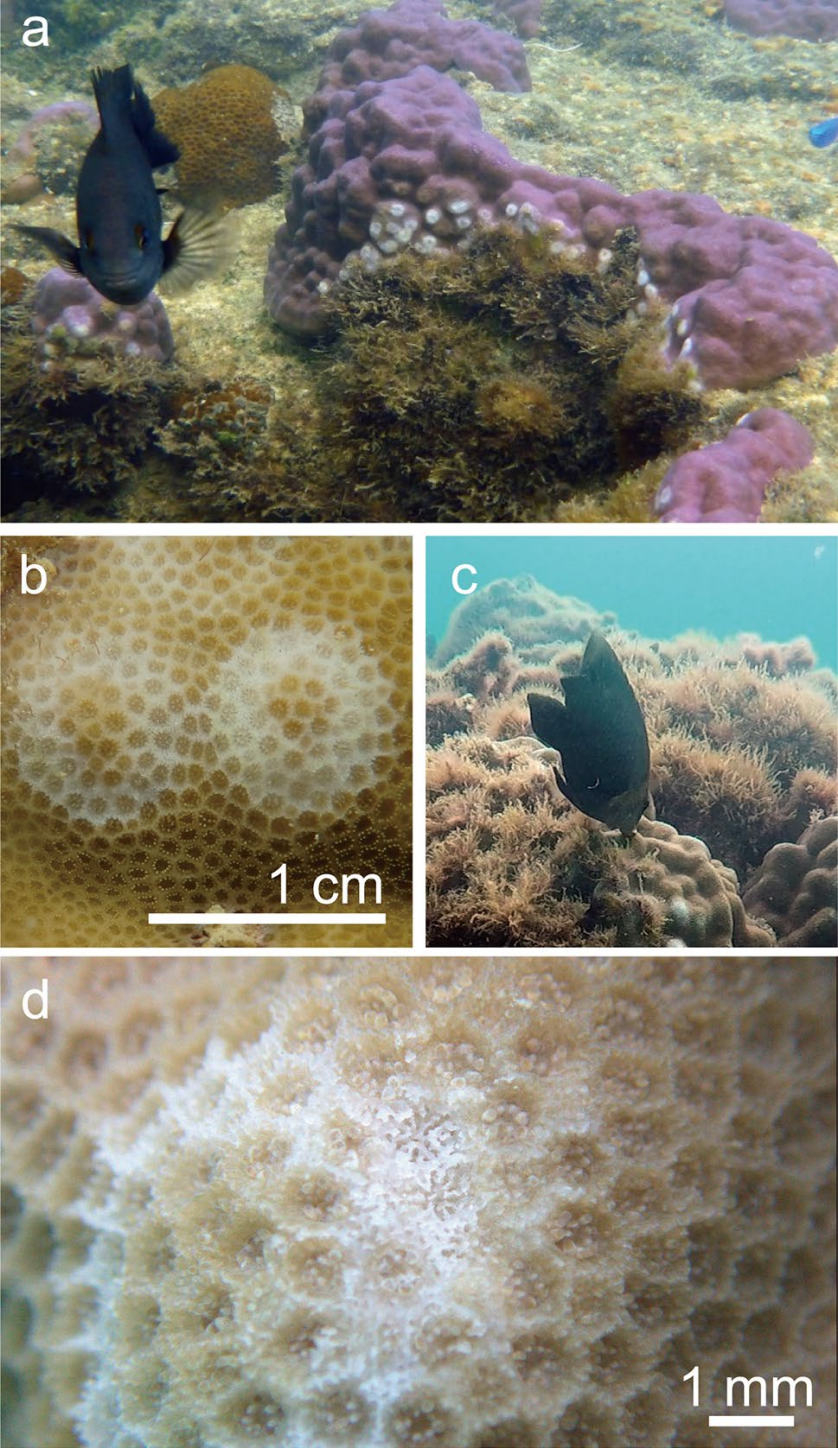
(**a**) White scars on live *Porites* around the territory of the herbivorous damselfish *Stegastes nigricans.* (**b**,**d**) Close-ups of white scars—bite lesions by *S. nigricans*. (**c**) *S*. *nigricans* biting live *Porites* adjacent to its territory.

Due to repeated mass coral bleaching under conditions of global warming, coral reefs are being degraded from a coral-dominated to an algae-dominated state^1–3^. Across the world, on some degraded reefs, turf algae have become the most abundant benthic group^4–8^. Turf algae and corals compete for space and light directly at coral–algal boundaries^9–11^. Herbivorous fishes contribute positively to the resilience of coral reefs in recovering from an algae-dominated state by removing algae, and also by enhancing coral recovery indirectly via the subsequent decrease in competition for space^12,13^. Conversely, herbivorous territorial damselfishes defend turf algae inside their territory and enhance the growth of turf algae^14,15^. Territorial damselfishes are abundant in number and their territories cover up to 70% of the reef substrate in some reef zones^14,16^. Therefore, they are able to occupy reef substrate and aid the expansion of turf algae, which prevents degraded coral reefs from recovering to a coral-dominated state^17–19^. Territorial damselfishes have sometimes been reported to bite coral tissues and disturb corals directly^20–22^. On Okinawa’s reefs, one territorial damselfish, *Stegastes nigricans*, maintains turf algae inside its territory as an algal farm on live coral colonies, such as massive *Porites* corals. On corals neighbouring these territories, white scars of about 1 cm in diameter have been observed. These white scars are thought to be bite lesions caused by the removal of coral tissue and upper layers of skeleton by territorial damselfishes, which possibly provides space for algal growth (Fig. 1^23,24^).

Territorial damselfishes are categorized into three guilds based on their farming strategies: intensive farmers, extensive farmers, and intermediate farmers^15,25^. *S. nigricans* is an intensive farmer that defends its small algal farms, which are dominated by a turf-forming *Polysiphonia* alga. The defensive behaviour of *S. nigricans* involves weeding and chasing away grazing fishes and invertebrates^15,26,27^. *S. nigricans* also chases away corallivorous fishes and maintains coral colonies inside its territories^28–30^. Extensive farmers defend large territories and mixed algal turfs comprising palatable filamentous algae and unpalatable algae^15,25^. Intermediate farmers do not weed but they keep mixed turfs as algal farms^15^. In this way, territorial damselfish species vary in their farming strategies and may have different impacts on coral reef resilience.

This study aims to reveal whether damselfishes cause the white scars on corals, and how these white scars contribute to the expansion and maintenance of their algal farms on live *Porites* colonies. We focus on an intensive farmer, *S. nigricans*, and an intermediate farmer, *S. lividus*, to compare different farming strategies. We conducted field surveys at three fringing reefs around Okinawa Island, Japan. We observed the coral–algal boundaries around and outside the territories of herbivorous damselfishes, the occurrence of white scars on corals adjacent to these boundaries, and algal colonization and coral recovery over the course of a 3-year period.

## Material and methods

### Study site

**Figure 2:**
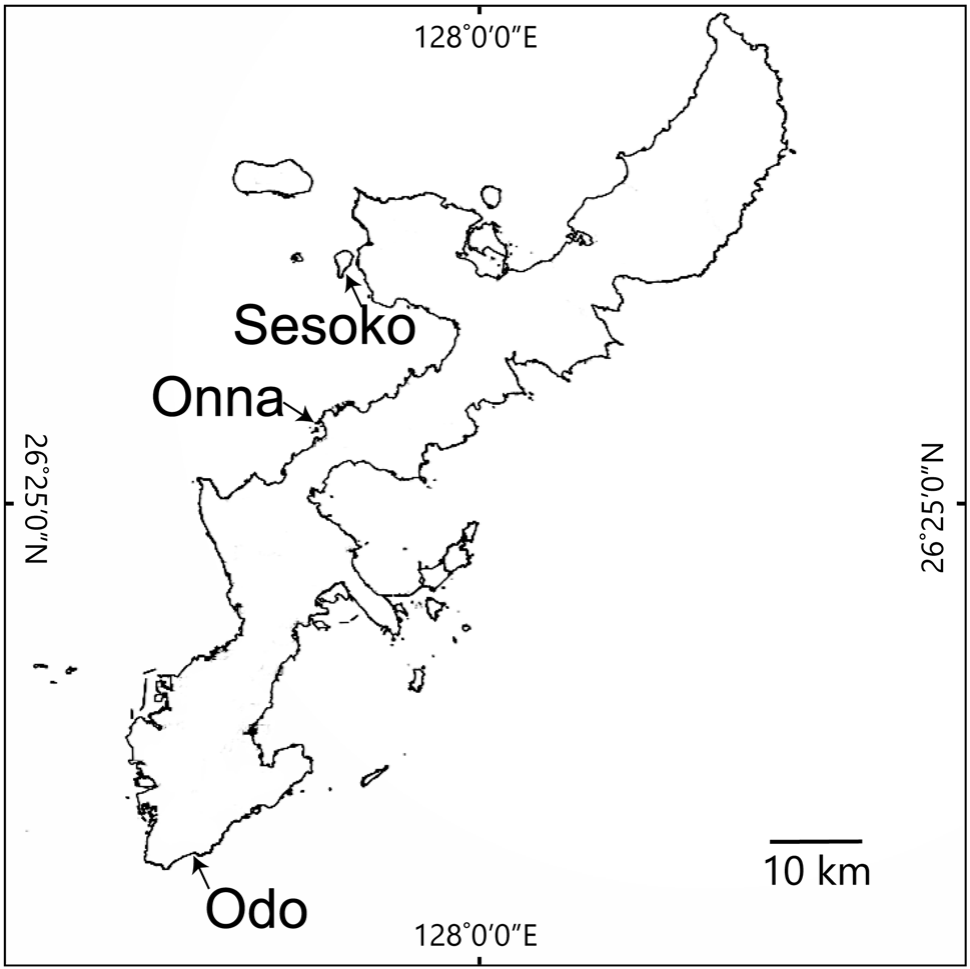
Map of Okinawa Island indicating the locations of our study sites on three fringing reefs.

We conducted field surveys at three fringing reefs around Okinawa Island, Japan: Sesoko, Onna, and Odo. The Sesoko site is on a reef slope 100 m away from the southeastern coastline of a small island, Sesoko Island, situated to the west of Motobu Peninsula, Okinawa Island (N 26° 38′ 08″, E 127° 51′ 55″; Fig. 2). The water depth at Sesoko is 2.4 ± 0.2 m (average ± SD) from the mean sea level (MSL). The Onna site is in a backreef moat with a reef crest 50 m away from the western coastline of Okinawa Island (N 26° 29′ 40″, E 127° 50′ 23″). The water depth is 1.5 ± 0.2 m from MSL. The Odo site is in a backreef moat with a developed reef flat 100 m away from the coastline at the southern end of Okinawa Island (N 26° 5′ 20″, E 127° 42′ 29″). The water depth is 2.1 ± 0.2 m from MSL. All three sites are little affected by terrestrial run-off, and coral coverage is relatively high*—*around 50 to 80%^31^. At all three sites, micro-atolls of massive *Porites* are the dominant habitat of territorial damselfishes.

### Study design and data collection

**Figure 3:**
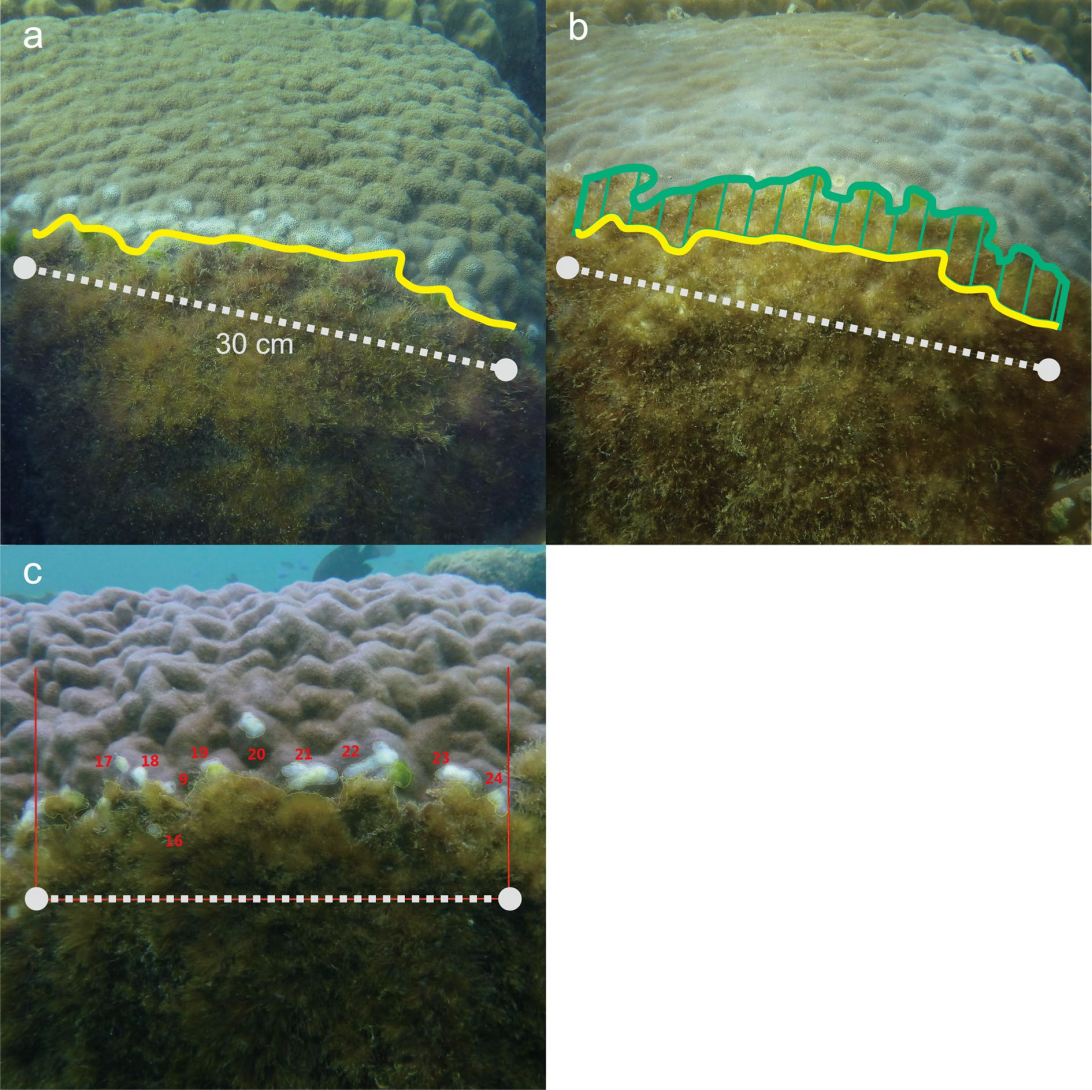
Example of a transect (grey dotted line) to observe the boundary between algal turf and adjacent live *Porites* coral. Yellow and green lines indicate the boundary on 17th June 2017 (**a**) and 4 weeks later (**b**), respectively. The green, hatched area denotes the algal turf expansion area (cm^2^), and we divided this area by the length of the transect (here 30 cm) and calculated the boundary movement (cm) for standardization among transects. White scars were marked one by one (number in red) and their area measured (**c**). At subsequent monitoring times, each white scar was checked to see whether it was covered by algae, re-covered by *Porites* tissue, or remained bare, and the areas of each cover type were measured.

We surveyed the territorial damselfish *S. nigricans* at all three sites. We also observed *S*. *lividus* at Onna, but it did not occur at the other two sites. We marked the territories of those damselfish that were established on massive *Porites* micro-atolls. We chose one to seven territories on each *Porites* micro-atoll and marked a 25-to-30-cm-long line transect parallel to the boundary between each territory and the adjacent live *Porites* corals from July to September 2015. Both ends of the transects were marked by driving concrete nails into the reef. As a control, we set line transects parallel to the boundaries between turf algae and adjacent live *Porites* corals where territorial damselfishes were absent. In total, 45 transects were set in 11, 18, and 16 territories of *S*. *nigricans* at Sesoko, Onna, and Odo, respectively, and six transects were set in six territories of *S. lividus* at Onna. Furthermore, 14, 5, and 10 transects were set in *Porites* colonies outside damselfish territories at Sesoko, Onna, and Odo, respectively (Supplementary Table S1). The number of observed territories of *S. lividus* was lower than that of *S. nigricans* because of a low density of *S. lividus* at our study sites. Line transects were monitored by taking pictures at a viewing angle parallel to the transect using a Nikon COOLPIX AW130 or W300 camera, 1, 2, and 4 weeks after setting the transects, and twice a year subsequently (Fig. 3a,b). Every time monitoring was carried out, territoriality (i.e., inside or outside territories of damselfishes) was determined by 15 min of observing whether the line transect was defended by territorial damselfishes or territorial damselfishes were absent. We counted the number of white scars in the images and measured their areas using ImageJ software. Each white scar was numbered and its coverage by algae and coral tissues, respectively, at each subsequent monitoring time was measured (Fig. 3c). The distance from the nearest coral–algal boundary was measured for each white scar. The area of white scars and coverage of white scars by algae and corals were added up for each line transect for analysis. We also measured the area of algal turf covering the coral–algal boundaries and then calculated the movement of the boundaries over time (Fig. 3; hereafter referred to as boundary movement) by dividing the change in algal turf area by the length of the line transect (25–30 cm).

### Data analyses

We analyzed the effects of territorial damselfishes on the shift in coral–algal boundaries using a generalized linear mixed model (GLMM), with the boundary movement for our 3-year observation period as the response variable, territoriality and site as fixed factors, and micro-atoll identity as a random factor. We also compared the effects of *S. nigricans* and *S. lividus* on boundary movement using a GLMM, with boundary movement as the response variable, damselfish species as a fixed factor, and micro-atoll identity as a random factor based on the data collected at Onna. We analyzed whether white scars would more likely be covered by algae or coral using a GLMM, with coverage (cm^2^) per transect as the response variable, coral or algae as a fixed factor, and micro-atoll and identity of the line transect as nested random factors. We analyzed the distance of each white scar from the territory boundaries of *S. nigricans* at Sesoko and compared white scars covered by algae with those re-covered by corals using a Mann–Whitney *U* test. We also analyzed the effect of white scars on shifts in coral–algal boundaries using a GLMM, with boundary movement as the response variable, area of white scars per transect as a fixed factor, and micro-atoll identity and boundary identity as random factors; boundary identity was nested within micro-atoll identity. Note that coral/algal coverage and areas of white scars per transect were divided by the length of each line transect (from 25 to 30 cm) for standardization. The GLMMs were run using the glmmML function in glmmML package 1.0.3 for R. All statistical analyses were conducted using R 3.5.1^32^.

## Results

### White scars on *Porites* at the coral–algal boundary and movement of the coral–algal boundary

**Figure 4:**
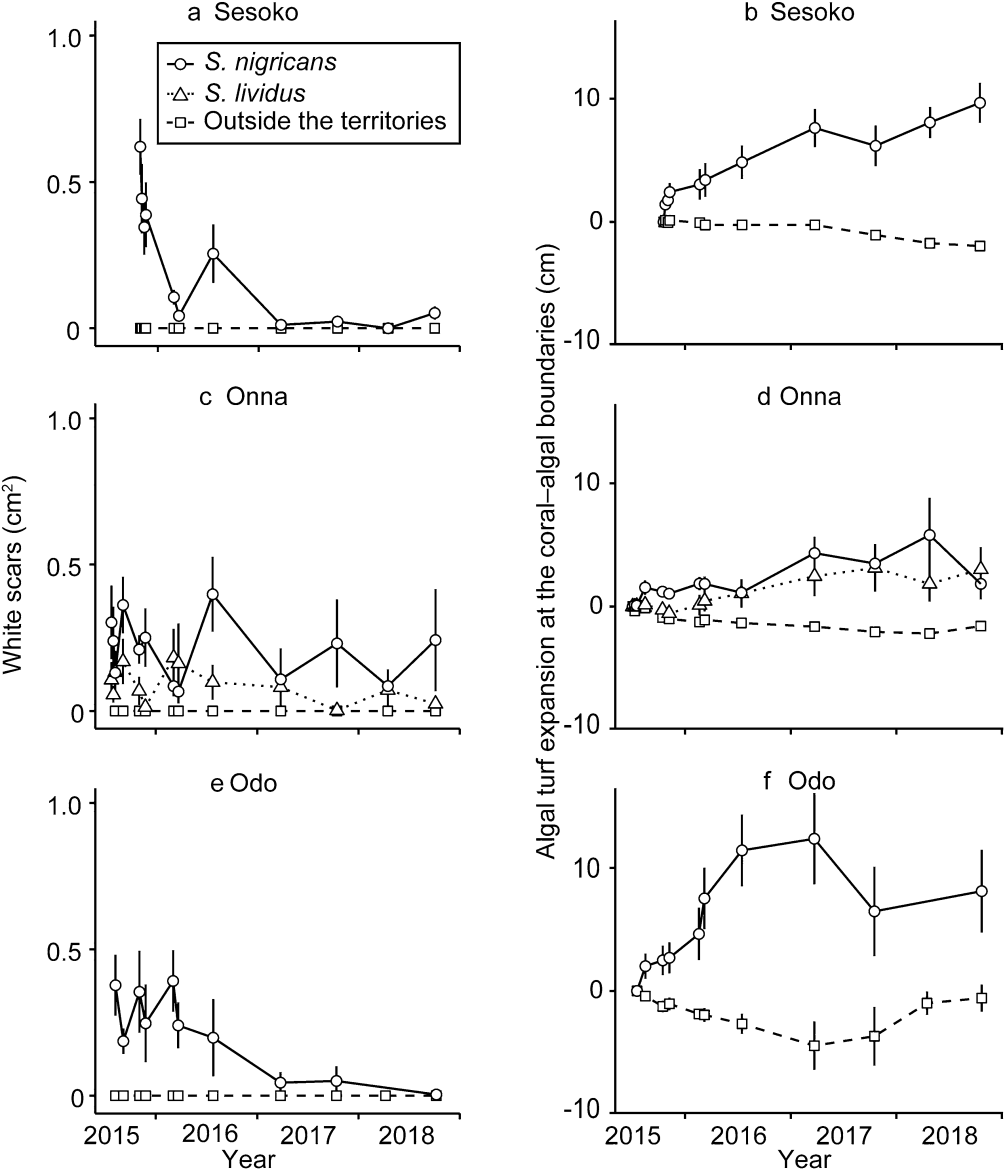
Area of white scars around coral–algal boundaries inside and outside damselfish territories at Sesoko (**a**), Onna (**c**), and Odo (**e**). Algal turf expansion at the coral–algal boundaries of damselfish territories and outside these territories at Sesoko (**b**), Onna (**d**), and Odo (**f**). The boundary and white scar area of each transect was observed repeatedly over the course of 3 years. For those transects where territoriality changed, summaries can be found in Supplementary Fig. S3. Error bars denote standard error.

White scars on *Porites* were found only adjacent to the territories of *S. nigricans* and *S*. *lividus* (Fig. 4a,c,e). The white scars were circular or doughnut-shaped, and had a mean area of 0.63 ± 0.34 (n = 385) and 0.74 ± 0.45 (n = 43) cm^2^ for *S*. *nigricans* and *S*. *lividus*, respectively; the size did not differ significantly between damselfish species (*t* test, *p* = 0.125).

At all study sites, the coral–algal boundaries around *S*. *nigricans* territories moved towards the coral, indicating expansion of the damselfish territories, which overgrew adjacent *Porites* (Fig. 4b,d,f). At the end of our observations in September 2018, the algal turf in *S*. *nigricans* territories had expanded significantly (Supplementary Table S2). Around *S*. *lividus* territories at Onna, the coral–algal boundaries also moved towards the coral side significantly compared to boundaries outside these territories (Fig. 4d, Supplementary Table S2). Furthermore, the movement of the coral–algal boundaries was not significantly different between territories of *S. nigricans* and *S. lividus* (GLMM, *p* > 0.05; Supplementary Table S3). Outside the damselfish territories, the coral–algal boundaries were relatively stable (Fig. 4b,d,f).

**Figure 5:**
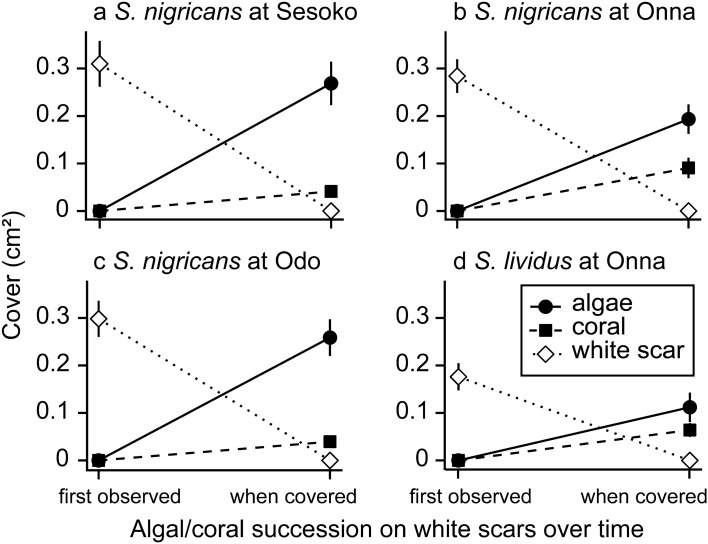
Succession on white scars of *Porites*, either covered by algae or re-covered by coral inside the territories of *Stegastes nigricans* at Sesoko (**a**), Onna (**b**), and Odo (**c**), and inside the territories of *S*. *lividus* at Onna (**d**). Error bars denote standard error.

**Figure 6:**
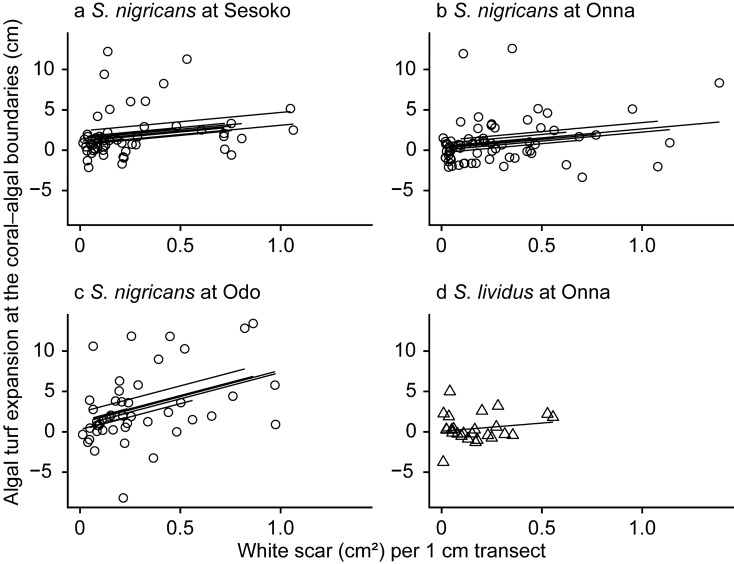
Relationship between the area of white scars and algal turf expansion at the coral–algal boundary of *Stegastes nigricans* territories at Sesoko (**a**), Onna (**b**), and Odo (**c**), and of *S*. *lividus* territories at Onna (**d**). The x axis is the area of white scars at a particular monitoring time, and the y axis is the movement of the coral–algal boundary between the monitoring time and the next. Each plotted point indicates a line transect at a particular monitoring time. For each transect individually, the area of white scars was summed and then this value divided by the length of the transect (25 to 30 cm). Lines are fitted based on a generalized linear mixed model.

Around *S*. *nigricans* territories at all three sites, significantly more white scars were covered by turf algae than re-covered by coral tissues (Fig. 5, Supplementary Table S4). White scars were more often covered by turf algae than re-covered by *Porites* corals when close to the coral–algal boundary (Mann–Whitney *U* test, *p* = 0.002, Supplementary Fig. S1). On the other hand, around *S*. *lividus* territories, white scars were covered by algae and corals to a similar extent. The coral–algal boundaries moved towards the coral more when the area of white scars was larger (Fig. 6, Supplementary Table S5) and when the algal coverage of the white scars was larger (Supplementary Fig. S2 and Table S6).

### Territory abandonment and the subsequent coral–algal boundary shift and occurrence of white scars

At Odo, Onna, and Sesoko, *S*. *nigricans* abandoned 8 of 16 (50%), 6 of 18 (33%), and 1 of 11 (9%) territories, respectively, during our 3-year observation period. When the coral–algal boundary was no longer inside a territory, the white scars disappeared and the corals overgrew the algae at the boundaries (Supplementary Fig. S3). By contrast, at Onna and Odo, four and three coral–algal boundaries, respectively, that started outside damselfish territories, were subsequently occupied by *S*. *nigricans*. Following this occupation by territorial damselfishes, white scars appeared and the coral–algal boundaries moved towards the coral side (Supplementary Fig. S3).

## Discussion

Our study showed that two species of territorial damselfishes*—S. nigricans* and *S. lividus—*bite *Porites* colonies adjacent to their territories, which causes white scars to appear on corals. Subsequently, these damselfish species expand their territories onto corals using algae-covered white scars as stepping stones. White scars appeared only at the coral–algal boundaries around the territories of *S. nigricans* and *S*. *lividus*. Furthermore, these white scars disappeared when damselfish territories were abandoned, but appeared when the coral–algal boundaries fell under damselfish territory. The white scars on *Porites* colonies were circular or doughnut-shaped, around 1 cm in diameter, and multifocal. This shape is characteristic and consistent with the reported shape of bite lesions of territorial damselfishes^23,24^. Our preliminary observations at Okinawa revealed that *S*. *nigricans* bit corals around its territories, which removed coral tissues and damaged coral skeletons (Fig. 1c,d). This damage causes white scars that provide turf algae with open space for colonization. After the experimental removal of some tissue of massive *Porites* in Okinawa, 70% of the lesions were covered by algae after 2 months^33,34^. When *Porites* tissue and partial skeletons were damaged, algae colonized the injured area and turf algae covered about 90% of this after 1 month; however, the *Porites* recovered completely after two more months in shallow reefs at our study sites^35^. Turf algae are susceptible to waves and herbivory, and corals frequently overgrow turf algae at the coral–algal boundaries when there are no damselfish on the shallow reefs of Okinawa^34^. In this study, white scars nearer to the territory boundaries tend to be covered by turf algae rather than re-covered by *Porites* corals, suggesting that damselfishes help turf algae to cover white scars by biting adjacent corals repeatedly to weaken the competitive ability of the corals^36^.

Both an intensive farmer, *S. nigricans*, and an intermediate farmer, *S. lividus*, promoted overgrowing of neighbouring live *Porites* colonies by turf algae in shallow fringing reefs off Okinawa. This was achieved by making white scars on corals, which the algae were able to colonize. However, extensive farmers have never been observed making white scars on corals, and they have less of an impact on corals within and around their larger territories in general^37,38^.

Territorial damselfishes can occupy reef substrate after coral death following disturbance, such as mass predation by the crown-of-thorns starfish *Acanthaster planci*, tropical cyclone, and mass coral bleaching, and sometimes increase their density on these disturbed reefs^16,39^. Increased territorial damselfishes expand the area of turf algae inside their territories on disturbed reefs, which prevents corals from recovering^18,19^. Conversely, these territories can actually provide a suitable substrate for coral recruitment and growth, especially for corals susceptible to corallivory, because these territories are defended against grazers and even corallivores by the damselfishes^28,29,36,40^. In fact, the number of coral recruits has been reported to be higher inside territories of *S. nigricans* than outside them on shallow backreefs at Moorea, French Polynesia^37^. Furthermore, species diversity of coral communities is reported as being higher inside territories of *S. nigricans* at Moorea, and inside territories of *S. lividus* on the reefs of Guam, although total coral coverage is limited inside these territories compared to outside^30^. Therefore, it is hypothesized that territorial damselfishes provide a nursery of seed populations for coral recovery after disturbance^30^. Our study showed that territories were frequently abandoned by *S. nigricans* during the 3-year observation period. Since, after abandonment of territories, adjacent *Porites* colonies recover their tissue and overgrow algal turfs, and corals recruited within territories do not continue to have their growth stifled under the control of damselfishes for a long time, the hypothesis above is partly supported. In this study, some territories on *Porites* micro-atolls were abandoned. In one study, over the course of a year on the reefs of Réunion, the boundaries of damselfish territories are shown to change and peripheral territories are abandoned because of continuous intraspecific competition and seasonal changes^41^. Our study site, Okinawa, is subtropical and has high seasonal variation in the energy demand of damselfishes and the productivity of algal farms^42,43^. These seasonal variations may cause the frequent abandonment of territories. Abandonment of territories may also be the mechanism that prevents a negative feedback cycle persisting for territorial damselfishes^44^. That is, territorial damselfishes destroy their own habitat by killing corals, because coral death enhances bioerosion of coral skeletons^45,46^, and bioerosion of coral skeletons results in the breakdown of the three-dimensional structure that is necessary for territories^44^. Continuous monitoring of (1) the densities of various damselfish species with intensive, extensive, and intermediate farming strategies, (2) the transfer of their territories, and (3) shifts in coral communities from before to after the abandonment of territories are all necessary to evaluate the effects that territorial damselfishes have on the resilience of coral reefs under repeated large-scale disturbances.

## Supplementary information

Supplementary Information.

## Data Availability

Raw data of the current study are available from the corresponding author on request.
