# Supplementary material for: Herbivorous damselfishes expand their territories after causing white scars on Porites corals
Source: Sci Rep. 2020 Sep 30;10:16172. doi: 10.1038/s41598-020-73232-8 (PMC7527513; doi:10.1038/s41598-020-73232-8)
Supplement: Supplementary file 1 — Supplementary Information. [file 41598_2020_73232_MOESM1_ESM.pdf]

Supplementary Information

Herbivorous damselfishes expand their territories after causing white scars on *Porites* corals

Hiroki Hata*, Shota Takano, Hiroyuki Masuhara

Hiroki Hata, Shota Takano, and Hiroyuki Masuhara have contributed equally to this study.

Graduate School of Science and Engineering, Ehime University, 2-5 Bunkyo, Matsuyama, Ehime 790-8577, Japan

Table S1. Numbers of transects and *Porites* micro-atolls studied inside and outside territories of *Stegastes nigricans* and *S. lividus*. Between one and seven territories were chosen on each *Porites* micro-atoll, and a transect was set on each territory. Between one and five transects were set on each *Porites* micro-atoll where territorial damselfish were absent (i.e. outside damselfish territories) and were set more than 1 m apart from each other.

Site	*S. nigricans*		*S. lividus*		Outside damselfish territories	
	No. Transect	No. *Porites* micro-atoll	No. Transect	No. *Porites* micro-atoll	No. Transect	No. *Porites* micro-atoll
Sesoko	11	8			14	5
Onna	18	5	6	2	5	2
Odo	16	4			10	3

Table S2. Results of generalized linear mixed models with the movement of coral–algal boundaries as the response variable, territoriality of *Stegastes nigricans* or *S. lividus* and site as fixed factors, and micro-atoll identity as a random factor. SE, standard error; DF, degrees of freedom.

Species	Value	SE	DF	t-value	p-value
S. nigricans					
(intercept)	-0.58	1.64	13	-0.35	0.73
territory of *S. nigricans*	8.68	2.32	13	3.75	0.0024
Onna	-1.01	3.66	13	-0.28	0.79
Sesoko	-1.38	1.97	13	-0.70	0.50
territory of *S.nigricans*: Onna	-5.25	4.27	13	-1.23	0.24
territory of *S.nigricans*: Sesoko	2.95	3.04	13	0.97	0.35
S. lividus					
(intercept)	-1.47	0.71	10	-2.08	0.06
territory of *S. lividus*	4.82	1.34	10	3.61	0.0048

Table S3. Results of generalized linear mixed models with the movement of the coral–algal boundaries at Onna as the response variable, damselfish species as a fixed factor, and micro-atoll identity and boundary identity as random factors; boundary identity was nested within micro-atoll identity. SE, standard error; DF, degrees of freedom.

	Value	SE	DF	t-value	p-value
(Intercept)	4.7	1.9	4	2.4	0.07
S. nigricans	-3.1	2.1	4	-1.4	0.22

Table S4. Results of generalized linear mixed models with coverage area as the response variable, site and whether coral or algae covered white scars as fixed factors, and micro-atoll identity and boundary identity as random factors; boundary identity was nested within micro-atoll identity. SE, standard error; DF, degrees of freedom; NS, not significant.

Species	Value	SE	DF	t-value	p-value
S. nigricans					
(intercept)	0.26	0.03	167	7.62	0.0000
algae/coral	-0.22	0.05	167	-4.76	0.0000
Onna	-0.07	0.04	159	-1.60	0.112
Sesoko	0.01	0.05	159	0.23	0.819
algae/coral: Onna	0.12	0.06	167	1.94	0.054
algae/coral: Sesoko	-0.01	0.06	167	-0.13	0.895
S. lividus					
(intercept)	0.11	0.02	25	4.62	0.0001
algae/coral	-0.05	0.03	25	-1.40	0.175

Table S5. Results of generalized linear mixed models with movement of coral–algal boundaries around the territories of *Stegastes nigricans/S. lividus* as the response variable, site and area of white scars per 1 cm transect as fixed factors, and micro-atoll identity and boundary identity as random factors; boundary identity was nested within micro-atoll identity. SE, standard error; DF, degrees of freedom.

Species	Value	SE	DF	t-value	p-value
S. nigricans					
(intercept)	1.02	0.78	138	1.31	0.19
Area of white scars	6.54	1.87	138	3.50	0.0006
Onna	-0.65	0.99	18	-0.65	0.52
Sesoko	0.28	1.01	18	0.27	0.79
Area of white scars: Onna	-4.32	2.32	138	-1.86	0.06
Area of white scars: Sesoko	-4.23	2.44	138	-1.73	0.09
S. lividus					
(intercept)	0.07	0.52	21	0.14	0.89
Area of white scars	2.02	2.33	21	0.87	0.39

Table S6. Results of generalized linear mixed models with the movement of the coral–algal boundaries around the territories of *Stegastes nigricans*/*S. lividus* as the response variable, site and area of algal cover on white scars per 1 cm transect as fixed factors, and micro-atoll identity and boundary identity as random factors; boundary identity was nested within micro-atoll identity. SE, standard error; DF, degrees of freedom.

Species	Value	SE	DF	t-value	p-value
S. nigricans					
(Intercept)	0.87	0.68	138	1.28	0.20
Algal cover	7.96	1.72	138	4.64	0.0000
Onna	-0.95	0.86	18	-1.10	0.29
Sesoko	0.34	0.89	18	0.38	0.71
Algal cover: Onna	-2.38	2.25	138	-1.06	0.29
Algal cover: Sesoko	-5.48	2.08	138	-2.63	0.009
S. lividus					
(Intercept)	-0.00	0.4	21	-0.01	0.99
Algal cover	3.73	2.08	21	1.79	0.09

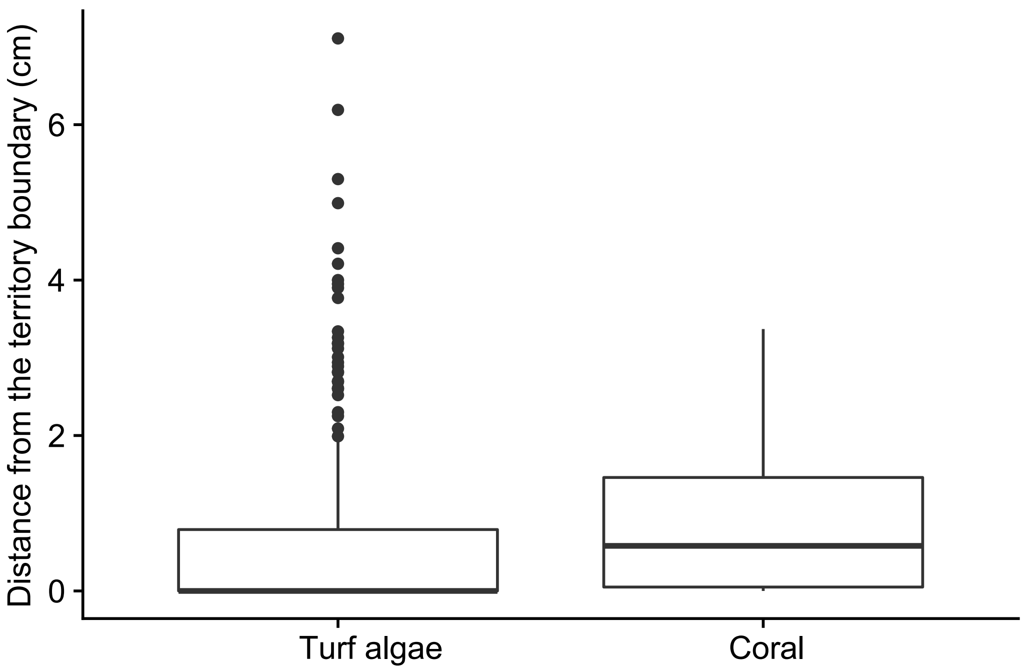

Figure S1. Distances from the territory boundary of *Stegastes nigricans* to the white scars that are covered by turf algae and those re-coverd by *Porites* corals.

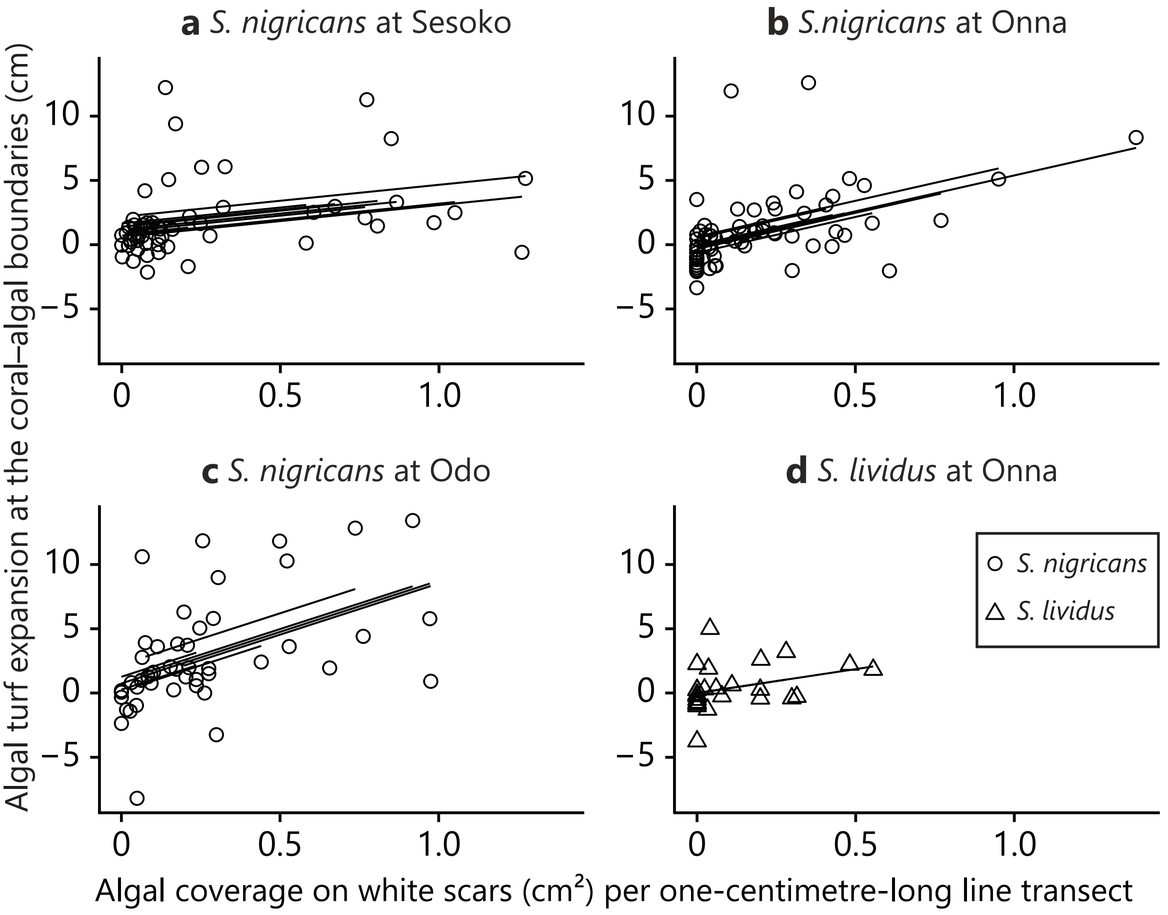

Figure S2. Relationships between algal coverage on white scars and algal turf expansion at the coral–algal boundaries of *Stegastes nigricans* territories at Sesoko (a), Onna (b), and Odo (c), and at the boundaries of *S. lividus* territories at Onna (d). Lines are fitted lines based on generalized linear mixed model.

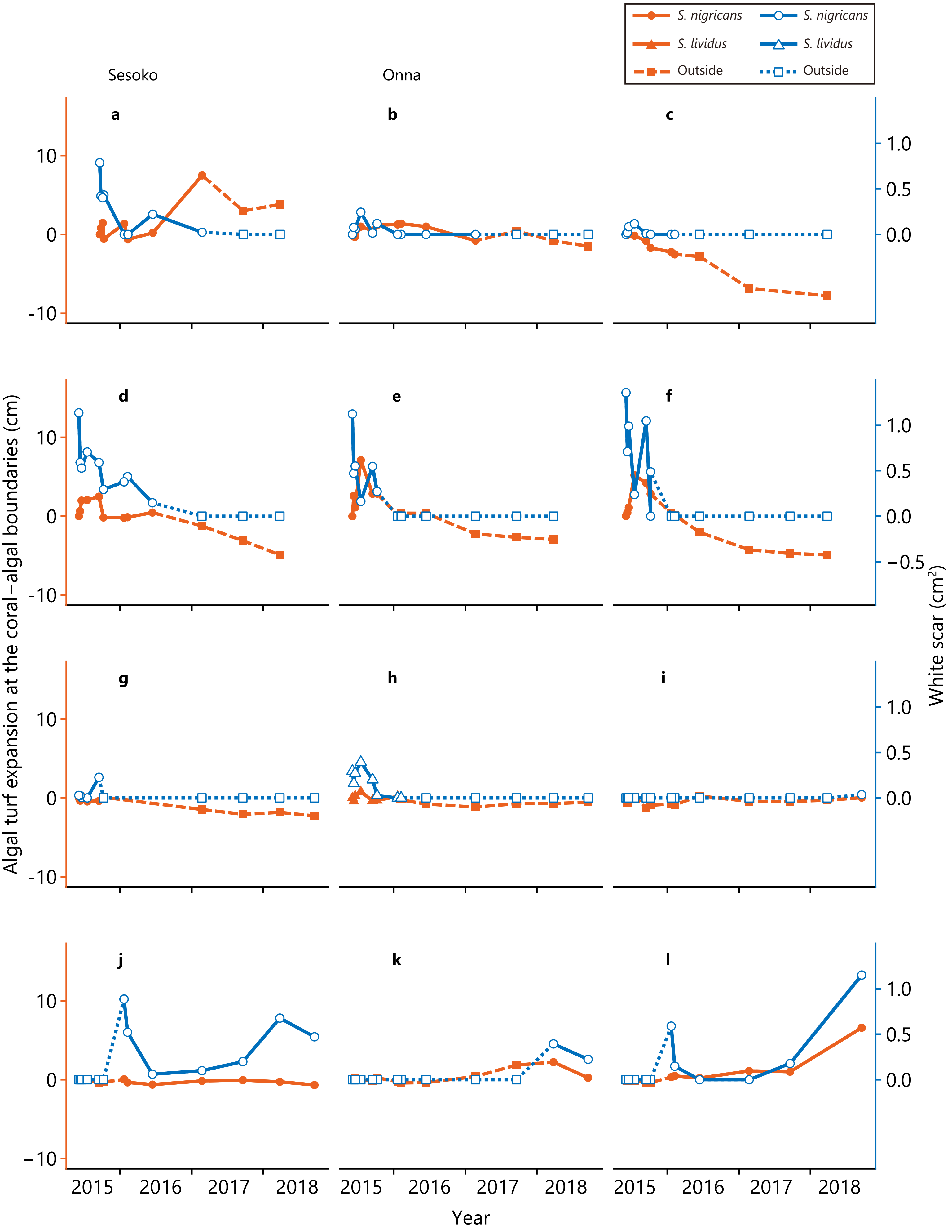

S. nigricans
S. lividus
Outside
S. nigricans
S. lividus
Outside
Sesoko
Onna
a
b
c
d
e
f
g
h
i
j
k
l
Algal turf expansion at the coral–algal boundaries (cm)
White scar (cm²)
Year
2015
2016
2017
2018
10
0
-10
1.0
0.5
0.0
−0.5

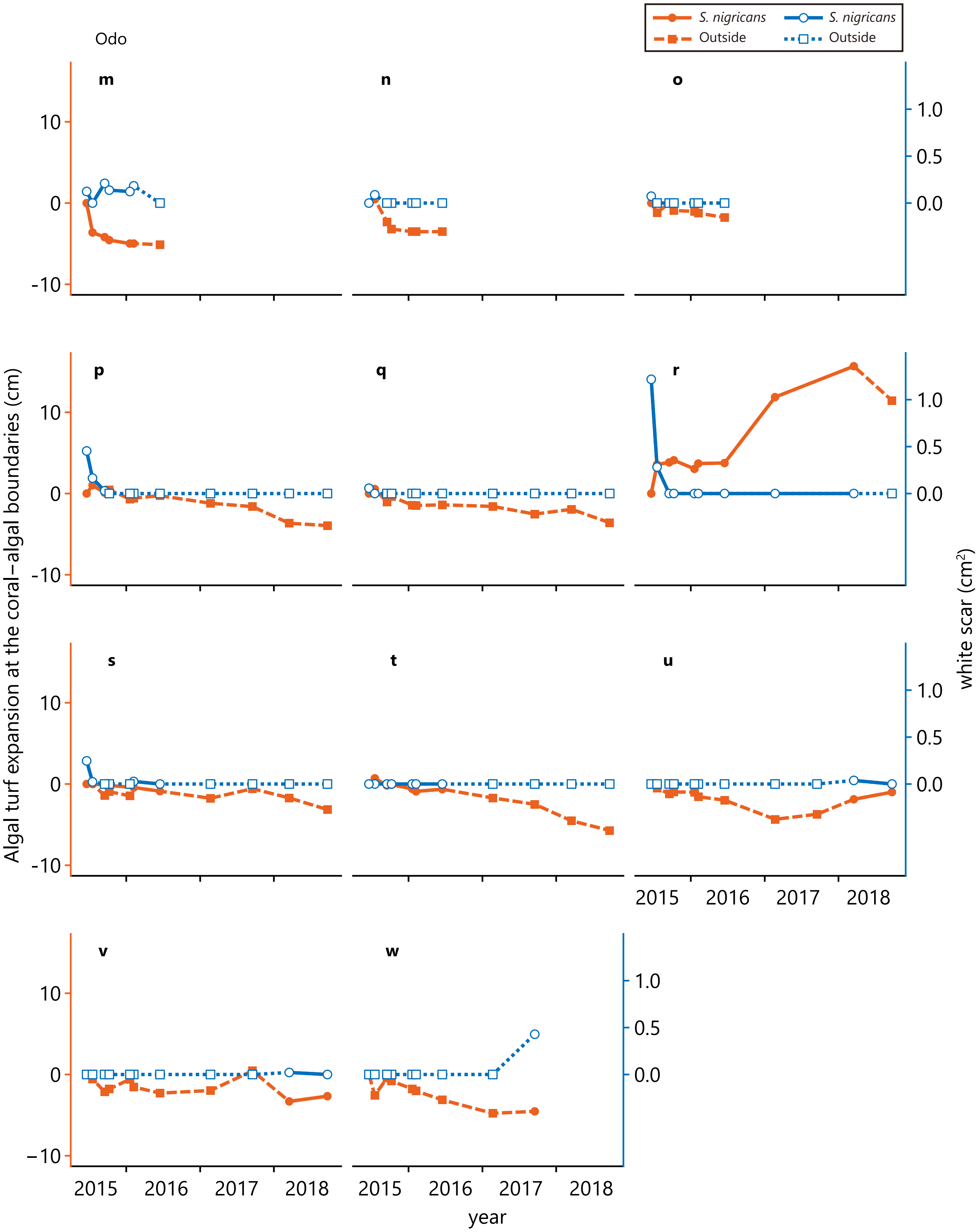

Figure S3. Algal turf expansion (red-line, left y-axis) and area of white scars (blue-line, right y-axis) at each coral—algae boundary of damselfish territory and outside the territory at Sesoko (a), Onna (b-l) and Odo (m-w).
